# Improving outcomes among young adults with type 1 diabetes: the D1 Now pilot cluster randomised controlled trial

**DOI:** 10.1186/s40814-022-00986-5

**Published:** 2022-03-08

**Authors:** Eimear C. Morrissey, Molly Byrne, Bláthín Casey, Dympna Casey, Paddy Gillespie, Anna Hobbins, Michelle Lowry, Elizabeth McCarthy, John Newell, Davood Roshan, Shikha Sharma, The D1 Now Young Adult Panel, Sean F. Dinneen

**Affiliations:** 1 Health Behaviour Change Research Group, School of Psychology, National University of Ireland, Galway, Ireland; 2 School of Medicine, National University of Ireland, Galway, Ireland; 3 Health Research Institute, University of Limerick, Limerick, Ireland; 4 School of Nursing and Midwifery, National University of Ireland, Galway, Ireland; 5 Health Economics & Policy Analysis Centre, National University of Ireland, Galway, Ireland; 6 CURAM, Science Foundation Ireland (SFI) Research Centre for Medical Devices, National University of Ireland, Galway, Ireland; 7 School of Mathematics, Statistics & Applied Mathematics, National University of Ireland, Galway, Ireland; 8 Centre for Diabetes, Endocrinology and Metabolism, Galway University Hospitals, Galway, Ireland

**Keywords:** Type 1 diabetes, Young adults, Behaviour change, Feasibility, Pilot randomised controlled trial

## Abstract

**Background:**

The D1 Now intervention is designed to improve outcomes in young adults living with type 1 diabetes. It consists of three components: an agenda-setting tool, an interactive messaging system and a support worker. The aim of the D1 Now pilot cluster randomised controlled trial (RCT) was to gather and analyse acceptability and feasibility data to allow (1) further refinement of the D1 Now intervention, and (2) determination of the feasibility of evaluating the D1 Now intervention in a future definitive RCT.

**Methods:**

A pilot cluster RCT with two intervention arms and a control arm was conducted over 12 months. Quantitative data collection was based on a core outcome set and took place at baseline and 12 months. Semi-structured interviews with participants took place at 6, 9 and 12 months. Fidelity and health economic costings were also assessed.

**Results:**

Four diabetes centres and 57 young adults living with type 1 diabetes took part. 50% of eligible young adults were recruited and total loss to follow-up was 12%. Fidelity, as measured on a study delivery checklist, was good but there were three minor processes that were not delivered as intended in the protocol. Overall, the qualitative data demonstrated that the intervention was considered acceptable and feasible, though this differed across intervention components. The agenda-setting tool and support worker intervention components were acceptable to both young adults and staff, but views on the interactive messaging system were mixed.

**Conclusions:**

Some modifications are required to the D1 Now intervention components and research processes but with these in place progression to a definitive RCT is considered feasible.

**Trial registration:**

ISRCTN (ref: ISRCTN74114336)

**Supplementary Information:**

The online version contains supplementary material available at 10.1186/s40814-022-00986-5.

## Key messages regarding feasibility

- What uncertainties existed regarding the feasibility?

The acceptability of the full D1 Now intervention package to both young adults living with T1D and diabetes staff was unknown. In addition, the feasibility of running a definitive cluster RCT in young adult diabetes clinics in Ireland was uncertain.

- What are the key feasibility findings?

With some modifications, two of the three D1 Now intervention components (the agenda-setting tool and the support worker) are acceptable and considered useful to both young adults living with T1D and diabetes staff. The process of running a definitive cluster RCT is also feasible, although some modifications to the current research processes are needed, including introduction of electronic data collection and engagement with local phlebotomy services to enhance the availability of HbA1c measurement.

- What are the implications of the feasibility findings for the design of the main study?

A cluster RCT of a refined D1 Now intervention is likely to be acceptable and feasible.

## Background

Young adults living with type 1 diabetes (T1D) have been highlighted as being at risk of lower engagement with self-management and higher blood glucose levels in comparison to younger and older people with the condition [1, 2]. Young adulthood can present as a challenging time for many, with pressures such as experimentation with drugs and alcohol, transitioning to higher education, new relationships and changing roles and responsibilities. Balancing the management of a complex chronic condition with the demands and unpredictability of young adulthood can be especially difficult [3] and is evidenced by relatively poor clinical outcomes including high blood glucose values [2] and descriptions of diabetes distress in this group [4, 5]. There is also a high rate of clinic non-attendance in this group, with relationships between young adults and healthcare professionals being cited as being an important factor in promoting clinic attendance [6]. Interventions are clearly needed to support young adults living with T1D and improve outcomes. A recent systematic review of interventions found that the quality of reported studies was poor, demonstrating a gap for a theory-based intervention informed by key stakeholder input to support and improve self-management and outcomes in young adults with T1D [7].

### The D1 Now intervention

D1 Now is a novel intervention, which has been developed using a systematic, theoretical, user-centred approach [3], the aim of which is to support self-management and clinic engagement and improve outcomes in young adults living with T1D. In Ireland, many hospital outpatient diabetes services offer “young adult” clinics aimed at delivering care to individuals aged approximately 18–25 who have transferred from paediatric or transition clinics [8]. The D1 Now intervention is delivered as an adjunct to usual care within these young adult clinics. Development of the D1 Now intervention was informed by a systematic review, qualitative research, expert consensus and was guided by the Behaviour Change Wheel [9]. This process has been described in detail elsewhere [10]. It consists of three components: a support worker, an interactive text messaging system and an agenda-setting tool. A stakeholder engaged approach has been central to the development of the intervention, whereby a public and patient involvement (PPI) panel, the D1 Now “Young Adult Panel”, participate as co-researchers in the study team. The panel consists of 10 young adults living with T1D who have contributed to all aspects of the research; the process of forming the D1 Now Young Adult Panel is described in more detail elsewhere [11].

### The D1 Now intervention components

#### The support worker

The support worker in the D1 Now intervention aims to provide continuity and build relationships between the young adult and their healthcare team. Briefly, the support worker is present at each young adult clinic appointment and ensures that the young adult has set an agenda for their appointment and that this agenda is followed through by the healthcare team. The support worker acts as an advocate for the young adult on the clinic day and, if appropriate, contributes to multidisciplinary team discussions for each young person. In addition, the support worker communicates with the young adult between clinic appointments on an individual basis.

This pilot cluster randomised controlled trial (RCT) explored the feasibility of two different models of incorporating the support worker into the diabetes team: (1) external support worker and (2) internal support worker. In the external support worker arm, the support worker was an additional member of staff who was hired for the purpose of the trial and was embedded in one intervention centre to join the existing diabetes team. In the internal support worker arm, a person who was an existing member of the diabetes clinic team (e.g. a nurse/doctor/dietician/psychologist) was upskilled on the role of the support worker by the research team and guidance on the role was available from the external support worker via phone or email as required. This distinction was considered important to investigate given the likely difference in resources required to fund an external support worker, when compared to an existing member of staff taking on the role. A detailed job description including role specification and duties of the support worker can be found in Appendix 1.

#### The interactive SMS-based messaging system

Florence is a software-based text messaging system developed in the UK that presents an easy-to-use interface for patients and clinicians with the aim of assisting people living with chronic disease [12]. Text-messaging “protocols” for monitoring a variety of conditions, such as type 2 diabetes, chronic obstructive pulmonary disease and cardiac failure have been developed [12, 13]. The D1 Now study team have adapted existing diabetes protocols on Florence for an Irish population of young adults living with T1D. The system operates by responding to health information sent and received by text message from the patient. Five types of text message protocols were used in the D1 Now pilot: blood glucose monitoring, alcohol safety, sick day rules, motivational messages and individualised protocols. Users can opt in or out of protocols to suit their needs. In the D1 Now pilot intervention arm centres, the internal or external support worker could liaise with young adults to set up individualised protocols.

#### The agenda-setting tool

**Fig. 1:**
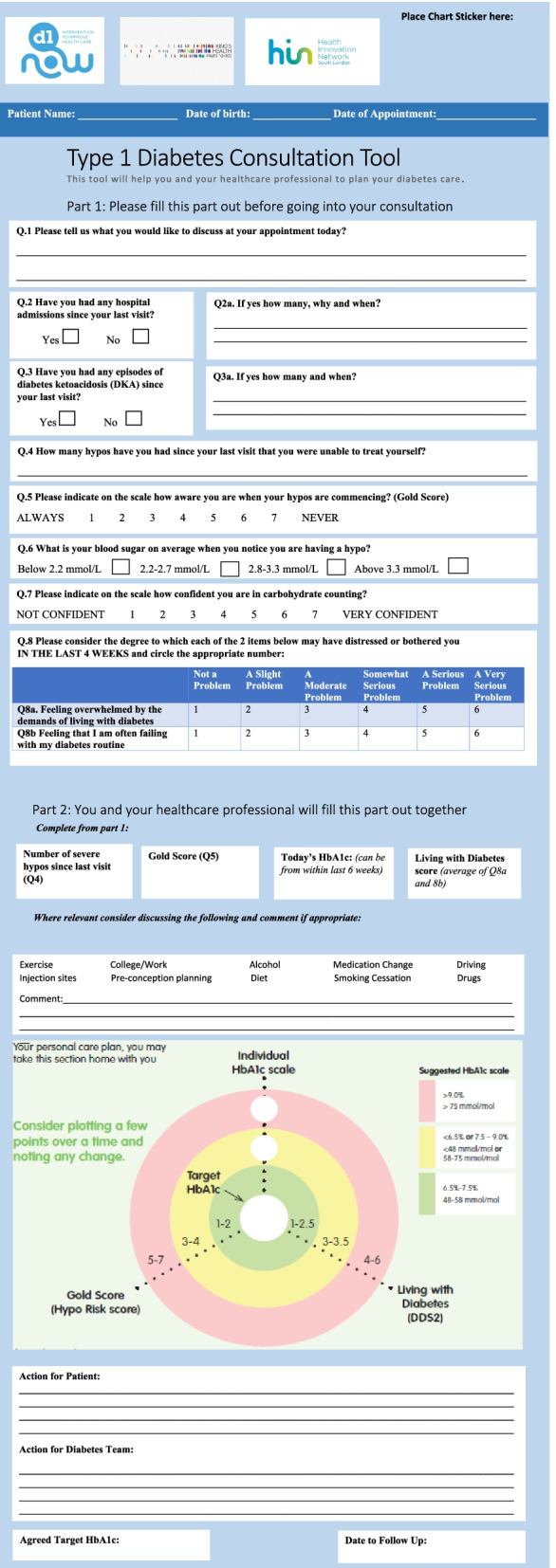
The D1 Now agenda-setting tool

The third intervention component is an agenda-setting tool which is used by the young adult before and during consultations and aims to improve the patient-clinician interaction to enhance shared decision-making. Through a scoping review of existing agenda-setting tools available internationally, the Type 1 Diabetes Consultation Tool (T1C) from the Health Innovation Network was chosen for use in D1 Now (Health Innovation Network—https://healthinnovationnetwork.com/projects/type-1-diabetes-consultation-tool-and-user-guide/). The T1C tool is specifically designed for the management of T1D and provides a holistic approach to care planning, bringing together a measure for psychological wellbeing (diabetes distress) as well as clinical results (Haemoglobin A1c (HbA1c) and hypoglycaemia unawareness). Diabetes distress is screened for using the Diabetes Distress Scale-2 (DDS-2) [14]. If the score on the DDS-2 is above 3 (out of a maximum of 6), the Type 1 Diabetes Distress Scale (T1-DDS) [15] is administered. The T1C tool enables the clinician to plot the results from the psychological and clinical measures on a dartboard-type chart prompting discussion on the relationship between these psychosocial and biomedical measures (Fig. 1). The T1C tool was adapted and refined for a young adult population by the D1 Now study team.

### The D1 Now intervention delivery

**Fig. 2:**
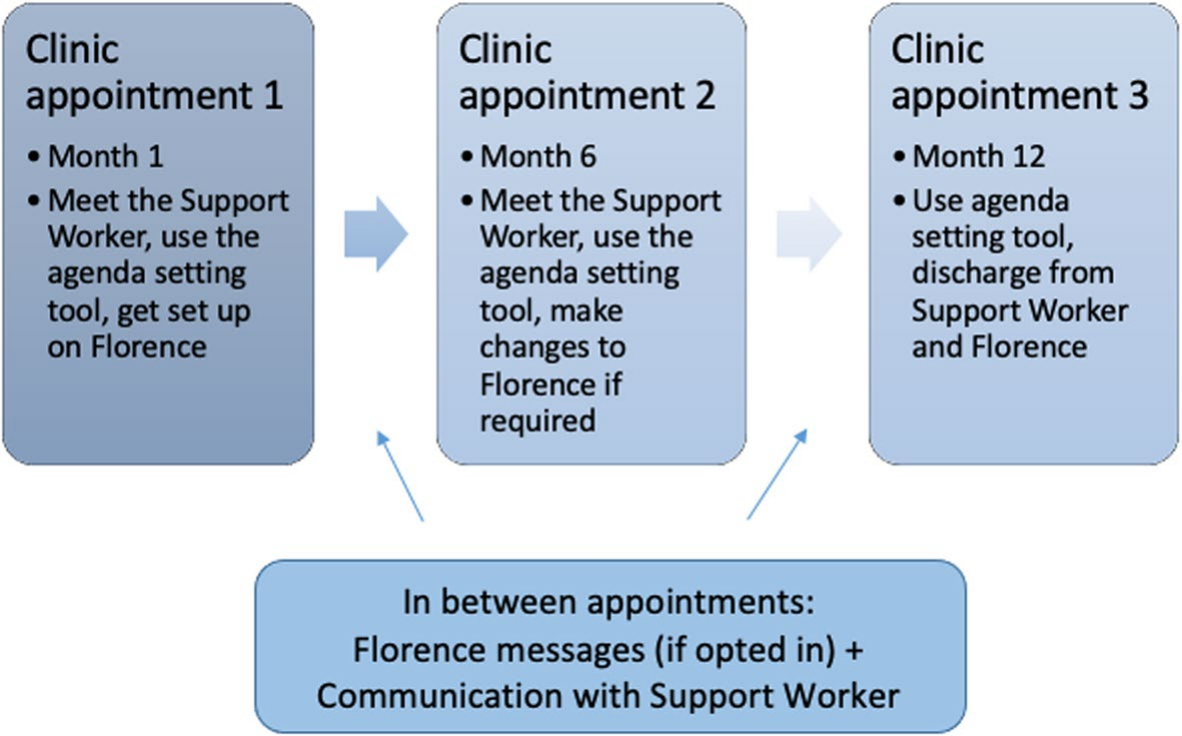
D1 Now intervention delivery timeline

The D1 Now intervention is delivered at a minimum of 3 clinic appointments over a 12-month period (see Fig. 2).

### Aims and objectives of the D1 Now pilot cluster RCT

The aim of the D1 Now pilot cluster RCT is to gather and analyse acceptability and feasibility data to allow us to (1) further refine the D1 Now intervention, and (2) determine the feasibility of evaluating the D1 Now intervention in a future definitive cluster RCT. Specifically, the D1 Now pilot study has the following objectives:

1. To investigate if the D1 Now intervention is feasible and acceptable to young adults living with T1D and diabetes healthcare staff;
2. To collect pilot qualitative and quantitative data to assess the feasibility of recruitment, retention and outcomes used;
3. To conduct a pilot health economic assessment of the D1 Now intervention;
4. To inform the sample size calculation, including the optimal number of diabetes centres (clusters) and young adults with T1D (participants), for a definitive cluster RCT.

## Methods

The pilot RCT has been registered (ISRCTN74114336) and a detailed protocol has been published [16]. Here, we briefly summarise our methods.

### Design

**Fig. 3:**
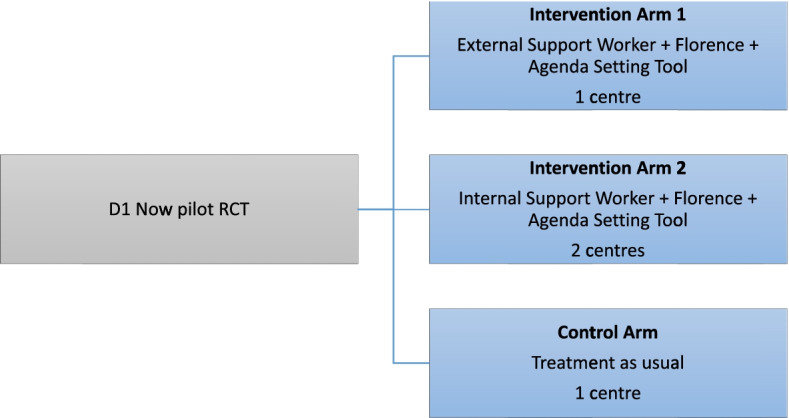
The D1 Now pilot RCT design

This was a cluster pilot RCT with two intervention arms and one control arm (see Fig. 3). The first intervention arm consisted of a single centre in which the D1 Now intervention was delivered with an external support worker (an additional member of staff employed for the purposes of the study). The second intervention arm consisted of two centres in which the D1 Now intervention was delivered with an internal support worker (an existing member of staff already employed within the diabetes team). The intention was to have the control arm consist of 2 centres delivering usual care however, as explained below, only one control arm centre took part.

### Inclusion criteria

Please see Table 1 for inclusion criteria for both diabetes centres and participants with T1D.

**Table 1:** Inclusion criteria

Inclusion criteria for diabetes centres	Inclusion criteria for participants
• A dedicated young adult clinic for people with type 1 diabetes • The young adult clinic should operate separately from other type 1 diabetes clinics that cater to young people, e.g. pump clinics and transition clinics • Eligible diabetes centres must have at least one full-time Diabetologist, Diabetes Specialist Nurse and Diabetes Specialist Dietician	• A confirmed diagnosis of type 1 diabetes for more than 12 months • Aged between 18 and 25 years on the date of recruitment • Participants on insulin pump therapy or using continuous glucose monitoring devices are eligible to participate • Participants must have access to a mobile phone.

### Sample size calculations

As this is a pilot study, a formal sample size calculation was not undertaken. One of the aims of the study is to generate the estimates needed for a sample size calculation for the definitive trial that will follow. We aimed to recruit 15–20 young adults in each pilot centre, a figure determined mainly by pragmatism.

### Recruitment

#### Centre recruitment

Five centres with a dedicated young adult clinic were recruited from a possible twelve on the island of Ireland [8, 16] (see protocol for details). However, due to delays in the research ethics application process and the onset of the COVID-19 pandemic, one of these centres (randomised to the control arm of the study) could not take part in the pilot RCT; therefore, four centres participated (three intervention and one control).

#### Participant recruitment

Study resources meant that only young adults who attended a diabetes clinic during October, November and December 2019 and January 2020 were approached and recruited into the study. Recruitment processes differed according to each centre due to varying ethical requirements. Currently, in Ireland, each hospital group has its own research ethics committee, rather than a national body. This can lead to different recommendations from different committees. In two of the centres (one in the external support worker arm, one in the internal support worker arm), researchers posted information sheets to all eligible young adults who were attending the October–January clinics. On the day of the clinic, the researcher met the young adult, and checked if they had received the information sheet. If so, the researcher answered any questions and consented the young adult into the study if they wanted to take part.

**Fig. 4:**
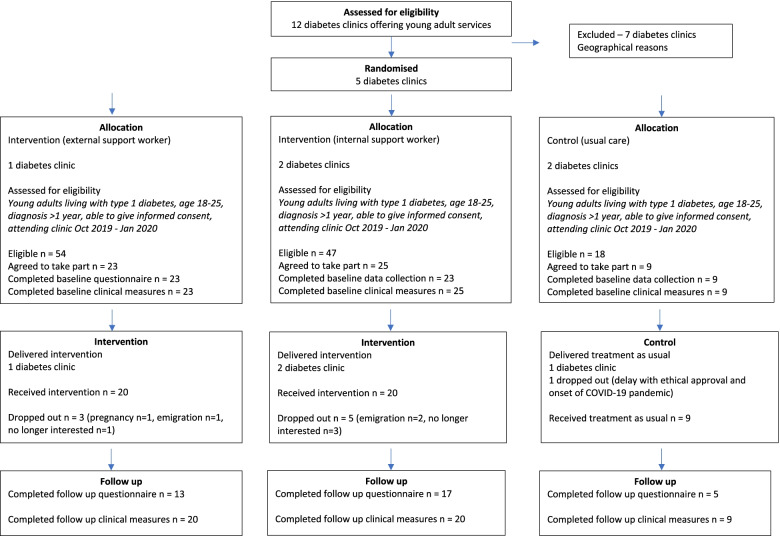
CONSORT flow diagram

In the two remaining clinics (one in the internal support worker arm and one in the control arm—both governed by the same ethics committee), a different recruitment process was required. Posting of information sheets prior to the clinic visit was not permitted. Instead, on the clinic day, a member of the clinic staff told eligible young adults about the study. If interested, the young adult filled in a “consent to be contacted by the research team” form. Their contact details were then sent to the research team who called the potential participant, after a minimum of 24 h had passed. If the potential participant was interested, study information, consent forms and baseline questionnaire had to be posted to and from the participant (see Fig. 4 for the CONSORT flow diagram).

### Randomisation procedures

One diabetes centre was randomised to the “D1 Now intervention with external support worker” arm, two diabetes centres was randomised to the “D1 Now intervention with internal support worker” arm and two diabetes centres were randomised to a usual care control (one of these centres subsequently left the study). The randomisation method used simple randomisation where a single sequence of random assignments was generated (with a fixed starting seed for reproducibility) [17]. The code (using R) needed to generate the randomisation sequence was written and executed by an independent statistician where the random number seed was recorded for reproducibility.

### Intervention procedures

The protocol required that the D1 Now intervention be delivered at a minimum of 3 clinic appointments during a 12-month period. The 2 intervention arms are described in detail in the study protocol [16]. Both centres randomised to the “D1 Now intervention with internal support worker arm” chose to upskill a Diabetes Specialist Nurse to the role of “internal support worker”. Participants in the control arm received usual care.

### Staff training

The support workers, internal and external, were provided with training on their roles and responsibilities by a member of the research team.

In addition, all young adult clinic staff in the D1 Now intervention arms were invited to attend D1 Now staff training, which was delivered by a member of the D1 Now research team using a pre-developed manual. These training sessions lasted for approximately 2.5 h.

### Data collection

Young adult data collection occurred at two time points during the trial:

- Baseline clinic visit (clinic appointment 1)
- End-of-study (12-month follow-up) visit (clinic appointment 3)

Paper-based self-report questionnaires were administered in the waiting room for those attending a face-to-face clinic appointment or were sent to participants by post. All other quantitative data were extracted from participants’ medical records by a clinical staff member.

#### Young adult questionnaires

The measures in Table 2 were included in the young adult questionnaire. The selection of outcome measures was informed by a recently published core outcome set for studies involving young adults with T1D [22].

**Table 2:** Patient outcome measures taken at baseline and 12 months

Construct to be measured	How was it measured?
Demographics	Self-report: gender, age, education status, occupation, duration of diabetes, co-morbidities, current insulin regimen, glucose monitoring method and other (non-insulin) medication.
Glycated haemoglobin (HbA1c)	Laboratory HbA1c within the last 3 months; if this was unavailable, the most recent point-of-care HbA1c result was used
Number of instances of diabetic ketoacidosis (DKA)	Medical record review and patient self-report; timeframe was over the past 12 months.
Number of instances of severe hypoglycaemia	Medical record review and patient self-report; timeframe was over the past 12 months.
Clinic engagement	This was operationalised as clinic attendance and was obtained from the clinic appointment administration system; timeframe was over the past 12 months.
Diabetes distress	Self-report: Measured using the Problems Areas in Diabetes-11 (PAID-11) scale [18].
Diabetes related quality of life	Self-report: Measured using the Audit of Diabetes Dependent Quality of life (ADDQOL) [19].
Diabetes related self-management	Self-report: Measured using the Diabetes Self-Management Questionnaire (DSMQ) [20]
Perceived level of control of diabetes	Self-report: Measured using the Diabetes Empowerment Scale-Short Form (DES-SF; 10) [21]

The young adult questionnaire also included questions on resource use (see “Health economic analysis”). Finally, it included a measure of acceptability and feasibility through an open textbox with the question “We would like to know what completing this questionnaire has been like for you, and what you have thought of the D1 Now study so far. Please write any comments that you have in the box below. All comments will be treated as confidential.”

#### Staff questionnaires

The staff questionnaire was administered at baseline only. The questionnaire gathered demographic information including profession and length of time working in diabetes.

#### Intervention fidelity

Staff filled in training delivery checklists after receiving intervention training. Support workers and young adults filled in a study delivery checklist after each clinic appointment. Examples of these checklists can be found in Appendix 3.

#### Quantitative evaluation of feasibility and acceptability

The items listed in Table 3 were measured to assess feasibility and acceptability.

**Table 3:** Quantitative evaluation of acceptability and feasibility of the D1 Now intervention and pilot cluster RCT methods

1. Recruitment of diabetes centres was assessed by documenting the number of invitations sent, the number of refusals and number of acceptances.	
2. Recruitment of participants was assessed by documenting the number of invitations sent, the number of initial responses, the number of follow-up phone calls required, the number of refusals and the number of acceptances.	
3. Loss to follow-up of participants was documented at every time point.	
4. Levels of missing data in completed questionnaires are reported.	
5. The comprehensibility and acceptability of all questionnaires were measured by asking participants how the questionnaires might be improved and how long they took to complete.	
6. Engagement with Florence is reported.	
7. The level to which the agenda-setting tool is used, in particular any missing sections, was documented.	

### Embedded qualitative component

A descriptive qualitative approach as described by Sandelowski [20] was used to explore the perceptions and experiences, as well as views on the feasibility and acceptability of the intervention, of young adults living with T1D (*n* = 16) and key healthcare staff (*n* = 10) participating in the D1 Now study. Qualitative description is particularly suitable for this work as “voices” of the participants are of critical importance, and it enables a largely unadorned or “data-near” description of their experiences [20]. This methodology allows the researcher to provide a descriptive summary of the facts of the case in everyday language. Young adult interviews took place at the midpoint of the study, around month 6, (*n* = 7) and at the end of the study, around month 12 (*n* = 9). All healthcare staff interviews took place at the end of the study, around month 12. In addition young adults (*n* = 3) and healthcare staff (*n* = 3) in the control group were interviewed, at month 9. Thematic analysis as outlined by Braun & Clarke [23] was used to analyse the data. This paper reports on the acceptability data from the qualitative work. More detailed results on participants’ experiences of the pilot RCT will be published separately.

### Health economic analysis

A pilot health economic assessment of the D1 Now intervention relative to usual care (control arm) was conducted. Resource use associated with delivery of the D1 Now intervention was measured and costed. In particular, resources relating to the external support worker, internal support worker, and other healthcare professional time input, Florence, the agenda-setting tool, educational sessions, consumables, materials, equipment and overheads were measured and costed. In addition, a form detailing the resources used by participants, including health service usage, insulin and insulin delivery usage was included in the questionnaires completed by the young adult participants at baseline and 12 months. Quality-adjusted life years (QALYs), the preferred outcome measure for economic evaluation, were estimated using the EuroQol EQ-5D-5L instrument at baseline and 12 months [24, 25].

### Statistical analysis

Data were entered into SPSS and descriptive statistics were used to summarise the findings. In order to calculate the sample size for a future definitive RCT, we used a sample size calculator designed for cluster randomised trials [26, 27].

### Progression criteria to a full RCT

The following pre-defined stop/go criteria were agreed to inform the decision on whether to proceed to a full-trial.

- Feasibility of recruitment of participants: At least 80% of study target recruited into the study
- Dropout rate: Less than 30% dropout of participants from intervention participation at 12 months in each group

A process for Decision-making after Pilot and feasibility Trials (ADePT) involves examining 14 methodological issues that are pertinent to feasibility research, such as recruitment, retention, randomisation, acceptability of processes and outcomes, intervention fidelity and estimation of costs [28]. A decision to progress will be decided based on the stop/go criteria, the findings of the ADePT process, findings from the qualitative research, and discussions with the study research team, advisory board and the Young Adult Panel.

### Ethics

Ethical approval was obtained from all centres—Beaumont Hospital Ethics (Medical Research) Committee (ref: 19/51), St James’s Hospital/Tallaght University Hospital Research Ethics Committee (ref: 2019-09 List 35 (13)), St Vincent’s University Hospital Research Ethics Committee (ref: RS19-031).

### Adaptations to the protocol

#### Impact of COVID-19 pandemic on intervention delivery

This pilot RCT took place from October 2019 to January 2021. The COVID-19 pandemic hit Ireland in early March 2020, with the majority of diabetes clinics moving to telephone or virtual clinic services by the end of that month. Some in-person clinics resumed in July–September 2020, depending on the centres. This impacted the delivery of the D1 Now intervention, which had been designed to be delivered in an in-person setting. Of the three intervention components, Florence was the least affected, as it continued to send text messages to participants’ phones. During the time of virtual clinics, the external support worker and one of the internal support workers moved their services online and contacted participants by phone, videocall or email. One internal support worker was redeployed to the COVID response for approximately 3 months. During this time it was not possible to deliver the support worker component of the D1 Now intervention in that centre. During the peak of the COVID pandemic, the agenda-setting tool was completed over the phone, rather than in person, where feasible. It is likely that this virtual delivery of the agenda-setting tool impacted participants’ experiences of the D1 Now intervention. This has been explored in the qualitative work.

#### Impact of COVID-19 pandemic on study procedures

As mentioned above, due to delays in the ethics application process and the onset of the COVID-19 pandemic, one of the centres allocated to the control arm was unable to take part in the pilot RCT.

The 12-month follow-up young adult data collection was impacted by the pandemic and use of virtual clinics. For baseline data collection 39/57 (68%) took place in the waiting room in advance of a clinic appointment and 18/57 (32%) through the post. Due to the move to virtual clinics and COVID-based restrictions, only 7/49 (14%) of the 12-month follow-up data collection occurred in the waiting room and 42/49 (86%) occurred through the post.

## Results

The completed CONSORT extension to pilot and feasibility trials checklist can be seen in Appendix 2.

### Sample characteristics

Demographic details of all young adult and staff participants are illustrated in Table 4.

**Table 4:** Participant baseline demographics (young adult participants: *n* = 57; staff participants: *n* = 30)

	Intervention arm 1	Intervention arm 2	Control arm
	External support worker	Internal support worker	Treatment as usual
**Young adult participants**	***n*** **= 23**	***n*** **= 25**	***n*** **= 9**
*Age (mean, SD)*	20.3 (1.8)	20.7 (1.8)	20.6 (1.9)
*Gender*			
Male	7	9	3
Female	16	14	6
*Marital status*			
Single	22	23	9
Cohabiting	1	–	–
*Age at T1D diagnosis (mean, SD)*	10.4 (4.9)	9.4 (4.4)	10.2 (4.7)
*Insulin therapy, mode of administration*			
Pen	15	14	7
Insulin pump	8	9	2
*Blood glucose monitoring, method*			
Finger prick	5	9	2
Continuous glucose monitor	1	8	1
Flash glucose monitor	17	6	6
*Previous participation in structured education course*			
Completed	3	2	5
Referred	6	4	2
Declined	4	6	1
Not offered	10	8	1
**Staff participants**	***n*** **= 7**	***n*** **= 11**	***n*** **= 12**
*Occupation*			
Administrator	1	2	3
Nurse	–	3	–
Diabetes Specialist Nurse	1	1	3
Dietician	1	1	2
Non-consultant Hospital Doctor	3	2	3
Consultant Endocrinologist	1	2	1

### Recruitment

The CONSORT diagram (Fig. 4) illustrates the flow of young adult participants through the study.

As described in the “Methods” section, two of the centres allowed recruitment to happen on site. This approach worked well, with approximately half of the eligible young adults opting to take part in the study. The other two centres required a longer method of recruitment, involving follow-up telephone calls and postage of study materials. This method of recruitment, with several additional barriers for the potential participant, led to a much lower recruitment rate.

### Retention

The attrition rate for the whole sample from baseline to follow-up was 12%. Three young adults from 23 (13%) dropped out of the external support worker arm, five dropped out of the internal support worker arm (20%), and there was no dropout from the control arm. Reasons for dropout included loss of interest, emigration and transfer of care (see the CONSORT flow diagram (Fig. 4) for more detail on this).

### Outcome measures

The outcomes measured are based on a core outcome set that was developed specifically for this group [22]. The core outcome set includes eight outcomes. Table 5 presents results for these outcome measures at baseline and follow-up for each arm of the study.

**Table 5:** Outcome measures at baseline and follow-up

	Baseline			Twelve months		
	Intervention Arm 1	Intervention Arm 2	Control Arm	Intervention Arm 1	Intervention Arm 2	Control Arm
	External support Worker (*n* = 23)	Internal support Worker (*n* = 25)	Treatment as usual (*n* = 9)	External support Worker (*n* = 20)	Internal support Worker (*n* = 20)	Treatment as usual (*n* = 9)
	*Completed responses n = 23*	*Completed responses n = 23*	*Completed responses n = 9*	*Completed responses n = 13*	*Completed responses n = 17*	*Completed responses n = 5*
PAID-11, mean (SD)	19.2(8.8)	19.5(8.0)	20.9(7.6)	17.4 (8.2)	16.1 (8.1)	16.8 (11.0)
DSMQ, mean (SD)	2.39 (0.8)	5.3 (1.2)	5.1(1.)	4.7 (1.3)	5.1 (1.7)	5.4 (0.87)
DES-SF, mean (SD)	3.5 (0.8)	3.8 (0.9)	3.7 (0.5)	3.9 (0.7)	3.6 (1.1)	3.7 (0.5)
ADDQOL, mean (SD)	− 3.3 (1.2)	− 3.8 (1.8)	− 4.1 (1.7)	− 4.7 (2.0)	− 3.4 (1.6)	− 3.3 (0.6)
HbA1c, mean (SD)	80.2 (17.9)	67.6 (20.2)	64.5 (12.3)	70.2 (16.0)	65.1 (12.1)	58.7 (16.1)
Clinic appointments attended: past 12 months, mean (SD)	2.1 (0.8)	2.6 (0.9)	1.8 (0.7)	3.4 (1.0)	2.6 (0.6)	1.6 (0.7)
Number of episodes of DKA in past 12 months	0: *n* = 20	0: *n* = 19	0: *n* = 6	0: *n* = 12	0: n = 16	0: *n* = 5
	1: *n* = 3	1: *n* = 4	1: *n* = 1	1: *n* = 1	1: *n* = 1	1: *n* = 1
	> 1: *n* = 0	> 1: *n* = 0	> 1: *n* = 2	> 1: *n* = 0	> 1: *n* = 0	> 1: *n* = 0
Number of episodes of severe hypoglycaemia in past 12 months	0: *n* = 21	0: *n* = 19	0: *n* = 6	0: *n* = 11	0: *n* = 15	0: *n* = 5
	1: *n* = 2	1: *n* = 2	1: *n* = 0	1: *n* = 1	1: *n* = 2	1: *n* = 1
	> 1: *n* = 0	> 1: *n* = 2	> 1: *n* = 3	> 1: *n* = 1	> 1: *n* = 0	> 1: *n* = 0

*SD* standard deviation

Four of these outcomes are self-report and were administered in the young adult questionnaire at baseline and month 12, alongside the health economics evaluation. The mean length of time reported to complete the questionnaire was 22 min (range—10 to 90 min). Feedback on these was extremely positive.

The questionnaire was very simple and concise to understand and fill out. (Female, age 19, diagnosed age 10, external support worker arm)

I enjoyed completing this questionnaire and it helped me realise somethings about my diabetes care management that I did not realise before. (Female, 19, diagnosed age 2, control arm)

The majority (*n* = 55/57; 96%) of questionnaires were completed at baseline. At time 2, 35/49 questionnaires (71%) were completed (8 young adults left the study—see CONSORT flow diagram). This lower rate of completion was likely caused by the requirement to send time 2 questionnaires by post to participants, rather than administer them during clinic attendance, due to COVID-19-related restrictions. The level of missing data from questionnaires was minimal across all measures.

For the clinical outcomes, including the number of episodes of DKA in the past 12 months, number of instances of severe hypoglycaemic in the past 12 months and clinic attendance, the completion rate at baseline and time 2 was 100%. The exception to this was HbA1c. For the purposes of this study, HbA1c measurement was operationalised as “laboratory plasma HbA1c within the last 3 months. Or, if this is unavailable, most recent point-of-care HbA1c test”. For baseline data collection we were able to obtain a laboratory HbA1c for 45 of the 57 participants (79%) from the medical notes. We obtained point of care HbA1c for eight of the remaining twelve. Problems with HbA1c measurement identified by clinic staff included the phlebotomy department being in a different part of the hospital campus and one clinic practice changing to once yearly lab HbA1c measurement. For time 2 data collection, the COVID-19 pandemic and associated restrictions impacted on HbA1c measurement, as many young adults did not attend clinics in person, but rather attended virtual clinics. Only 19 of 49 participants (39%) had a recent HbA1c measurement that could be used for analysis.

### Stop/go criteria

The stop/go criteria for recruitment of participants (80% of study target) was met, as 57 young adults were recruited which represents 95% of the required target of 60. The stop/go criteria for participant dropout rate (no more than 30% dropout of participants at 12 months in each group) was also met.

### Sample size calculation for future definitive trial

We based the sample size calculation on our data, supplemented with data from another study [29] and anticipated the mean HbA1c difference between the 2 arms of the study to be 4 mmol/mol at 1-year follow-up. Based on a standard deviation of difference in HbA1c of 10.5 and an intra-class correlation coefficient (ICC) of 0.0115, we estimated that 420 patients from 12 clusters (35 patients from each cluster) would be required to detect a 4 mmol/mol difference in HbA1c with 90 percent power. To maintain the power of our study to detect a clinically important difference and allowing for dropouts, we plan to increase our total sample size to 492 patients (41 patients from each cluster).

### Health economics analysis

The methods developed and implemented for the conduct of the pilot health economics analysis proved to be feasible and acceptable to study participants. In terms of the cost data generated in the pilot, the implementation cost of the D1 Now intervention was estimated at €1281 per young adult for the internal support worker arm and €1804 per young adult for the external support worker arm. In terms of other healthcare resource usage and costs, summary statistics are presented in Tables 6 and 7. In terms of total costs, the mean cost per patient for the internal support worker arm was estimated at €3755 (SD: 1762), the external support worker arm at €3944 (SD: 1489), and the control arm at €2217 (SD: 2706). In terms of health economic outcomes, summary statistics for EQ-5D-5L utility scores and QALYs at 12 months are presented in Table 6. The mean QALYs per patient was estimated for the internal support worker arm at 0.9 (SD: 0.1), the external support worker arm at 0.9 (SD: 0.2), and the control arm at 0.8 (SD: 0.2).

**Table 6:** Resource use and EQ-5D-5L estimates at baseline and follow-up

Variable/time point	Baseline						Follow-up: 12 months					
	Intervention arm 1		Intervention arm 2		Control arm		Intervention arm 1		Intervention arm 2		Control arm	
	***N*** = 23		***N*** = 23		***N*** = 9		***N*** = 23		***N*** = 23		***N*** = 9	
	External support worker		Internal support worker		Treatment as usual		External support worker		Internal support worker		Treatment as usual	
	*Mean (SD)*		*Mean (SD)*		*Mean (SD)*		*Mean (SD)*		*Mean (SD)*		*Mean (SD)*	
**Resource item**												
GP visits	2.5	(2.5)	2.4	(1.9)	4.1	(3.9)	1.00	(1.6)	1.2	(1.2)	1.9	(1.7)
Practice Nurse visits	0.0	(0.0)	0.4	(1.0)	0.1	(0.3)	0.3	(1.1)	0.7	(1.4)	0.0	(0.0)
Psychologist visit	1.5	(4.1)	0.8	(1.8)	0.0	(0.0)	1.7	(6.4)	2.5	(6.6)	6.5	(15.9)
Diabetes Specialist Nurse visit	2.2	(1.7)	2.2	(1.9)	2.2	(2.4)	0.7	(1.4)	1.7	(1.4)	0.7	(0.8)
Dietician visit	1.3	(2.7)	0.9	(1.3)	1.4	(1.1)	0.3	(0.6)	0.2	(0.6)	0.7	(0.8)
Diabetes Day Centre	1.9	(1.4)	2.3	(1.3)	1.6	(0.8)	2.2	(1.6)	1.2	(1.0)	0.8	(1.0)
Outpatient visits	0.8	(3.0)	0.3	(0.6)	0.9	(1.5)	0.5	(1.6)	0.4	(0.7)	0.8	(1.5)
Inpatient days	0.1	(0.5)	0.6	(1.6)	0.1	(0.3)	0.0	(0.0)	0.1	(0.2)	0.0	(0.0)
A&E visits	0.4	(0.7)	0.4	(0.7)	1.3	(1.4)	0.1	(0.3)	0.4	(0.6)	0.5	(0.5)
SA insulin*	15	(65%)	18	(78%)	6	(67%)	11	(48%)	16	(70%)	6	(67%)
LA insulin*	12	(52%)	11	(48%)	6	(67%)	6	(26%)	7	(30%)	4	(44%)
Ultra-LA insulin*	0	(0%)	0	(0%)	1	(11%)	0	(0%)	0	(0%)	1	(11%)
Insulin pump*	8	(35%)	10	(43%)	2	(22%)	6	(26%)	8	(35%)	2	(22%)
Multiple daily injections*	13	(57%)	12	(52%)	7	(78%)	8	(35%)	8	(35%)	4	(44%)

*GP* general practitioner, *A&E* accident and emergency, *SA* short acting, *LA* long acting

Completeness of data:

External support worker: *Baseline*—0% missing data for GP visits, 4% for practice nurse visits, 4% for Psychologist visit, 4% for Diabetes Specialist Nurse visits, 0% for Dietician Visit, 4% for Diabetes Day Centre visits, 22% for outpatient visits, 4% for hospital inpatient nights, 0% for A&E visits, 9% for insulin therapy, 9% insulin device used and 0% for EQ-5D-5L

External support worker: *Follow-up*—39% missing data on GP visits, Practice Nurse visits, Psychologist visit, Diabetes Specialist Nurse visits, Dietician Visit, Diabetes Day Centre visits, outpatient visits, hospital inpatient nights, A&E visits insulin therapy, insulin device used, EQ-5D-5L and QALYs

Internal support worker: *Baseline*—4% missing data on GP visits, 9% Practice Nurse visits, 9% for Psychologist visit, 9% for Diabetes Specialist Nurse visits, 13% for Dietician Visit, 9% for Diabetes Day Centre visits, 30% for outpatient visits, 9% for hospital inpatient nights, 9% for A&E visits, 4% for insulin therapy, 4% insulin device used, and 0% for EQ-5D-5L

Internal support worker: *Follow-up*—26% missing data on GP visits, Practice Nurse visits, Psychologist visit, Diabetes Specialist Nurse visits, Dietician Visit, Diabetes Day Centre visits, hospital inpatient nights, A&E visits, insulin therapy, insulin device used, EQ-5D-5L and QALYs. 30% missing data for outpatient visits

Treatment as usual: *Baseline*—0% missing data for GP visits, Practice Nurse visits, Psychologist visit, Diabetes Specialist Nurse visits, Dietician Visit, Diabetes Day Centre visits, outpatient visits, A&E visits, hospital inpatient nights, insulin therapy, insulin device used and EQ-5D-5L

Treatment as usual: *Follow-up*—33% missing data on GP visits, Practice Nurse visits, Psychologist visit, Diabetes Specialist Nurse visits, Dietician Visit, Diabetes Day Centre visits, hospital inpatient nights, A&E visits, for insulin therapy, insulin device used, EQ-5D-5L and QALYs 56% missing data for outpatient visits

*Annualised costs were estimated and applied for insulin, pump and multiple daily injections

**Table 7:** Costs, EQ5D scores and QALY estimates at baseline and follow-up

Variable/time point	Baseline						Follow-up: 12 months					
	Intervention arm 1		Intervention arm 2		Control arm		Intervention arm 1		Intervention arm 2		Control arm	
	***N*** = 23		***N*** = 23		***N*** = 9		***N*** = 23		***N*** = 23		***N*** = 9	
	External support worker		Internal support worker		Treatment as usual		External support worker		Internal support worker		Treatment as usual	
	*€* *Mean (SD)*		*€* *Mean (SD)*		*€* *Mean (SD)*		*€* *Mean (SD)*		*€* *Mean (SD)*		*€* *Mean (SD)*	
**Resource item**												
GP visits	125.00	(122.98)	119.05	(94.51)	205.56	(194.37)	50.00	(77.83)	60.00	(62.22)	95.83	(84.29)
Practice Nurse visits	0.00	(0.00)	14.64	(41.58)	4.56	(13.67)	11.71	(43.83)	28.94	(57.55)	0.00	(0.00)
Psychologist visit	148.50	(407.33)	77.79	(177.49)	0.00	(0.00)	169.71	(635.01)	247.5	(648.24)	643.50	(1576.25)
Diabetes Specialist Nurse visit	28.66	(22.21)	28.43	(24.57)	28.43	(31.46)	9.08	(18.07)	21.61	(18.37)	8.75	(10.71)
Dietician visit	81.71	(172.42)	58.50	(82.08)	90.28	(68.52)	20.00	(40.98)	15.29	(36.55)	43.33	(53.07)
Diabetes Day Centre	259.12	(192.89)	318.53	(175.65)	219.11	(106.32)	294.67	(215.69)	165.75	(128.95)	113.33	(133.71)
Outpatient visits	110.5	(407.15)	34.00	(78.52)	120.89	(208.98)	68.00	(218.32)	51.00	(97.76)	102.00	(204.00)
Inpatient days	133.29	(446.06)	540.16	(1469.15)	103.67	(311.00)	0.00	(0.00)	54.88	(226.29)	0.00	(0.00)
A&E visits	109.64	(178.52)	95.71	(175.45)	357.33	(379.01)	19.14	(71.63)	110.35	(165.72)	134.00	(146.79)
SA insulin	347.03	(196.59)	297.08	(148.92)	301.90	(174.53)	271.23	(194.44)	343.25	(184.88)	313.08	(198.59)
LA insulin	341.42	(137.91)	407.16	(180.22)	320.61	(69.76)	337.74	(167.94)	420.61	(170.97)	392.81	(125.39)
Ultra-LA insulin	0.00	(0.00)	0.00	(0.00)	396.48	(0.00)	0.00	(0.00)	0.00	(0.00)	352.43	(0.00)
**Insulin pump**	3439	(0.00)	3439	(0.00)	3439	(0.00)	3439	(0.00)	3439	(0.00)	3439	(0.00)
**Multiple daily injections**	612	(0.00)	612	(0.00)	612	(0.00)	612	(0.00)	612	(0.00)	612	(0.00)
**Total healthcare cost**	2550.56	(1358.01)	3006.50	(1844.00)	2680.69	(1674.14)	2139.59	(1488.82)	2474.12	(1761.75)	2216.51	(2705.10)
**D1 Now intervention cost**							1803.57	(0.00)	1281.41	(0.00)	€0.00	(0.00)
**Total costs**							**3943.59**	**(1488.82)**	**3755.12**	**(1761.75)**	**2216.51**	**(2705.10))**
**Health outcome**												
EQ-5D-5L Score	0.8	(0.2)	0.9	(0.1)	0.8	(0.2)	0.9	(0.2)	0.8	(0.2)	0.8	(0.2)
QALY gained							0.9	(0.2)	0.9	(0.1)	0.8	(0.2)

Completeness of data:

External support worker: *Baseline*—0% missing data for GP visits, 4% for Practice Nurse visits, 4% for Psychologist visit, 4% for Diabetes Specialist Nurse visits, 0% for Dietician Visit, 4% for Diabetes Day Centre visits, 22% for outpatient visits, 4% for hospital inpatient nights, 0% for A&E visits, 9% for insulin therapy, 9% insulin device used and 0% for EQ-5D-5L

External support worker: *Follow-up*—39% missing data on GP visits, Practice Nurse visits, Psychologist visit, Diabetes Specialist Nurse visits, Dietician Visit, Diabetes Day Centre visits, outpatient visits, hospital inpatient nights, A&E visits insulin therapy, insulin device used, EQ-5D-5L and QALYs

Internal support worker: *Baseline*—4% missing data on GP visits, 9% Practice Nurse visits, 9% for Psychologist visit, 9% for Diabetes Specialist Nurse visits, 13% for Dietician Visit, 9% for Diabetes Day Centre visits, 30% for outpatient visits, 9% for hospital inpatient nights, 9% for A&E visits, 4% for insulin therapy, 4% insulin device used, and 0% for EQ-5D-5L

Internal support worker: *Follow-up*—26% missing data on GP visits, Practice Nurse visits, Psychologist visit, Diabetes Specialist Nurse visits, Dietician Visit, Diabetes Day Centre visits, hospital inpatient nights, A&E visits, insulin therapy, insulin device used, EQ-5D-5L and QALYs. 30% missing data for outpatient visits

Treatment as usual: *Baseline*—0% missing data for GP visits, Practice Nurse visits, Psychologist visit, Diabetes Specialist Nurse visits, Dietician Visit, Diabetes Day Centre visits, outpatient visits, A&E visits, hospital inpatient nights, insulin therapy, insulin device used and EQ-5D-5L.

Treatment as usual: *Follow-up*—33 % missing data on GP visits, Practice Nurse visits, Psychologist visit, Diabetes Specialist Nurse visits, Dietician Visit, Diabetes Day Centre visits, hospital inpatient nights, A&E visits, for insulin therapy, insulin device used, EQ-5D-5L and QALYs. 56% missing data for outpatient visits

### Intervention fidelity

Intervention components were generally very well adhered to but a few key issues were highlighted in the study delivery checklists and qualitative interviews. The first of these was a lack of formal training on the agenda-setting tool given to junior doctors who rotated into the clinic while the study was ongoing. This meant that when a young adult was seen by a junior doctor who had not been working in the clinic at the beginning of the study, the tool may not have been used correctly.

Another issue related to the order in which the young adult saw healthcare professionals during the visit. According to the intervention protocol, a young adult should be seen first by the support worker, then by clinic staff and again by the support worker at the end. However, on busy clinic days, some young adults were seen first by a doctor.

Finally, inconsistent administration of the full (17-item) T1-DDS questionnaire when a young adult scored above 3 on the DDS-2 was identified as a protocol deviation. In some cases the support workers felt that this was not a practical use of time, especially when sources of distress had been identified in previous appointments. Not using the T1-DDS meant that the support worker would have more time to discuss support options with the young adult.

### Intervention acceptability

Overall feedback was generally very positive; however, views on acceptability differed according to each intervention component. The agenda-setting tool and support worker were liked by both young adults and staff, although staff had some concerns around the resourcing of the support worker. Views on Florence were mixed. This is outlined in detail below.

#### Acceptability of D1 Now to young adult participants

Many young adults say that they were very happy to take part in diabetes research generally, as they feel much research overlooks the T1D young adult group. As a result, taking part in this research was very acceptable to them, with one participant (female, 18, diagnosed age 10, interviewed at 12 months, external support worker arm) describing it as a “privilege”.

In terms of the D1 Now intervention itself, again, feedback was generally very positive. Many participants spoke about being grateful to have had the chance to use the intervention components.

Would recommend anyone to take part on this study/process going forward as I found it very helpful. [Support Worker] has been brilliant and I can’t put into words how thankful I am to her, from minute one she has been great and so easy to communicate with, so thanks very much for allowing me to partake in this study. I truly believe it was been very beneficial for me now and going forward. (Male, 19, diagnosed age 5, comment in open-ended textbox on questionnaire, External support worker arm)

When the intervention components were delved into in more detail it became clear that the agenda-setting tool and the support worker were favoured over Florence by almost all participants. Young adults spoke about how using the agenda-setting tool made them feel more in control in busy clinic appointments, empowered them to speak about issues that they may be feeling shy or embarrassed about, kept their appointments more focused and was something they would like to keep using in the future. The tool itself was generally fully completed, with no one part consistently skipped over by participants. Common areas for discussion requested by participants included diabetes technology, motivation and exercise.

After the first appointment that I had, the sheet of the things I would like to get out of the meeting with the doctor [the agenda setting tool] was one thing that sort of writing it down, and going into the meeting, the meeting does be nearly 100 miles an hour and you have different things going on, but having it written down in front of you was something I picked up on straight away that I would love to do more often like. (Male, 21, diagnosed age 18, interviewed at 6 months, External support worker arm)

The young adults were also generally very supportive of the inclusion of a diabetes distress measure in the agenda-setting tool (DDS-2) [14]. Many emphasised the importance of mental health in diabetes self-management and felt their clinical care often focused heavily on the physical aspects of diabetes. Several young adults recalled the agenda-setting tool sparking a conversation around diabetes distress that may not have occurred otherwise.

I really like that question. Yeah, that mightn’t have been something you were, you know, you’d discuss unless say maybe you had a problem. As in, it was, yeah, it was definitely nice to be asked that and that would kind of lead to a bit of a conversation around it. Yeah, because that isn’t something discussed in the kind of mental health side of diabetes, definitely isn’t probably discussed as much as it should be in clinics. (Female, 20, diagnosed aged 9, interviewed at 12 months, Internal support worker arm)

The support worker—both external and internal—was also received very well by the young adults. They considered it a very acceptable addition to their diabetes care as their clinic appointments became more structured and holistic in nature and a sense of continuity was provided.

I think [support worker] was a very good idea because usually how it works in Irish hospitals is that you go there and usually every time, there’s a different doctor or it can happen that it’s like that. And I kind of like to always have the same person so you can check up and follow-up and see if you’ve had any progress or not. And it’s a lot easier if you always work with the same person. In the study, it’s actually possible because you have the Support Worker who always works with you. So it’s the same person every time. So that’s a really good thing about it. (Female, 22, diagnosed age 4, interviewed at 12 months, Internal support worker arm)

But like compared to what normally the visits would be, it [support worker] kind of just gave them a bit more support and it made it kind of easier going in kind of knowing what you’re going to talk about or knowing what’s going to come up. More so than… And it kind of made it less results-based. So it wasn’t solely based around my HbA1c. It was kind of based around how I felt how I was getting on and so forth, like stuff like that so. (Male, 18, diagnosed age 10, interviewed at 12 months, External support worker arm)

The final intervention component, Florence, received mixed reactions. Approximately 75% of young adult participants signed up to receive the text messages. Those who did not sign up indicated that they thought the messages would become annoying. Some of those who did sign up reported that they did find the messages annoying, and disengaged quickly. Sixteen percent of those who signed up to receive the messages asked their support worker to turn Florence off. The others engaged in passive usage, where the interactive functions of Florence (e.g. texting back blood glucose values) were rarely used.

I did [Florence] for a little while and then they just kind of annoyed me. … So I kind of just got to a point where I just ignored them. (Female, 22, diagnosed age 14, interviewed at 12 months, External support worker arm)

A number of young adults felt that they already had access to adequate diabetes technology and didn’t need another system.

I am on like the pump but I have the sensor and stuff so that kind of tracks what my blood sugars are anyway So I felt it [Florence] was kind of like an additional step that monitored information that I was already giving. (Female, 19, diagnosed age 12, interviewed at 6 months, Internal support worker arm)

#### Acceptability of D1 Now to staff

Most staff who took part in the study had positive overall feedback and were glad to have taken part. Some mentioned how the young adults in their services seemed to appreciate research being done in this area and in their clinic. They also felt that the young adult participants seemed to have benefitted from using the intervention components and had more satisfactory clinic appointments as a result.

In my opinion, the young adults probably had a more meaningful consultation in terms of, I think they got more exposure to their support person, which in our environment was a nurse educator. And I think that the questionnaire [agenda setting tool], was a really useful way to target and focus and include the patient perspective and priorities and to encourage the patient to have priorities and a perspective for their visit. (Consultant Endocrinologist, Internal support worker arm)

Staff reflected on using the D1 Now intervention during the usual busy clinic. Meeting the support worker and using the agenda-setting tool required extra time and this sometimes disrupted the flow of the clinic. Some staff changed around their appointment lists as a means of overcoming this.

The only difficulty we had at the start was space and the flow of the clinic because I'm afraid our clinics were sort of three hour clinics, and they were sandwiched in between one clinic and another clinic, so you have to fit in that defined time. So at the start of the study we did have issues with space and the numbers that the [Support Worker] could see within one clinic and just the flow of the clinic. (Consultant Endocrinologist, External support worker arm)

When discussing the individual intervention components, the agenda-setting tool was universally praised. Consultants felt it improved their consultations with young adults by allowing the usual conversation to become more two sided. Other staff liked that it had a section for the clinician to make a plan as well as the young adult, so that the goal-setting process felt more collaborative.

Really liked it. I thought it improves the quality of the consultation and it ensures that the consultation is a two-way process addressing the concerns and priorities of both sides, rather than a doctor’s perspective of what needs to be discussed. (Consultant Endocrinologist, Internal support worker arm)

So I did really like the idea of it and also it focused us more I think as clinicians to come up with an actual plan at the end and to be quite specific with our targets and things and what was expected. So yeah, I think it had positives for both sides. (Dietician, External support worker arm)

The inclusion of the DDS-2 on the agenda-setting tool was appreciated by most staff. Several interviewees mentioned that by using the DDS-2, conversations around distress that might previously have been avoided were now easier to initiate. It also meant that distress that may have been previously undetected was caught and discussed.

So in the past if I didn’t have the tool I would have said to someone how are you feeling, how are you getting on and they would have said “grand” I would have gone “grand” but now if you have a value where the score is high you're no longer accepting “grand” you're sort of saying “listen you know you scored high here are you okay?” (Consultant Endocrinologist, External support worker arm)

All staff who worked with a support worker found it to be a very valuable resource. They all talked about how positive it was for the young adults to have a continuous point of contact in the clinic. They also saw the benefit in young adults having a person to talk to who was not a usual member of their healthcare team. This was particularly the case for the staff who worked with the external support worker.

We would love to have that element as a permanent element in our unit. (Consultant Endocrinologist, External support worker arm)

Some challenges were identified for the internal support workers. Both staff members who volunteered to take on this role were Diabetes Specialist Nurses. It was acknowledged that taking on this role meant some time away from their usual duties, which put an extra workload on their colleagues.

We had an internal support worker on this site, and that was one of our Diabetes Nurse Specialists, so I suppose the impact it had on me probably personally was if she was needing to do some of that work, I was picking up maybe a bit more of the other diabetes work” (Dietician, Internal support worker arm)

While this worked reasonably well for one team, the other was already severely under-resourced and a Diabetes Specialist Nurse taking on additional duties caused strain. Some staff members felt that in order for a support worker role to work well, the role would need to be a protected resource, akin to the external support worker.

But you know, it’s an additional piece of work in an already over-stretched unit. So it needs to be ring fenced, secured, protected resource (Consultant Endocrinologist, Internal support worker arm)

However, there were some advantages to an existing staff member taking on the role of a support worker. Both felt that their clinical skills came in very useful during meetings with young adults, and this meant that meetings could achieve several different goals. On the other hand, the external support worker was trained in mental health and this was also considered valuable by staff, as it meant that she was comfortable in talking about diabetes distress with both young adult participants and the other healthcare staff. She was able to provide training on diabetes distress to the team and many felt the benefit of this upskilling.

So in a lot of ways, I feel like my background in mental health was quite crucial in terms of the role. I suppose even the nature of the D1 Now intervention, like the agenda-setting tool, having a specific screening for distress, being able to have the conversation about distress and I suppose looking at the impact of that, with a background in mental health I suppose helped bring that to a place where actually we can do something with this. (External support worker)

Challenges were also identified with the external support worker role. As this is a new role in the diabetes service, there was some clarity lacking in the exact day-to-day duties involved.

So the first couple of weeks in particular, just trying to figure out what is it that I’m here to do? So that was kind of a lot of kind of what I felt at the start was I suppose the definition of the role, part of this year of this intervention was to figure out what it is a support worker does. So part of that required me kind of feeling my way in terms of what the role was about. (External support worker)

Another key challenge was putting a new person, in a new role, into an existing diabetes team with a busy young adult service. A significant amount of flexibility was required in order to successfully navigate established systems and relationships, and encourage the change in practice required to successfully deliver the intervention.

I suppose, you know, sometimes things have been done a certain way for a long time and this is quite a new approach and it is showing us a different way of looking at things, lots of different things to consider, and there can be a bit of a reluctance to commit to that. (Dietician, External support worker arm)

The reactions to Florence by the staff were mixed, similar to that of the young adults. The concept of an interactive text messaging system being outdated in the world of diabetes technology was brought up by several staff. One support worker pointed out that a lot of the functionalities provided by Florence could probably be provided by the participant’s personal devices already.

And if I was to describe it, like the old VCR compared to like something much more digital now. (Internal support worker, Diabetes Specialist Nurse)

I guess I’d have mixed feelings about how useful young people found Florence based on the level of engagement and whether or not actually it may be slightly out of date at the moment as a technology tool, that there probably are more kind of readily available apps in a young person’s phone or kind of calendar tools where reminders can be set up that maybe from a cost perspective, maybe something like Florence, I don’t know if it’s value for money in terms of an intervention component necessarily. (External support worker)

#### ADePT process

ADePT involves examining 14 methodological issues that are pertinent to feasibility research. Table 8 presents these methodological issues, a mapping of these to the research questions and relevant findings.

**Table 8:** Summary of findings for the Decision-making after Pilot and feasibility Trials (ADePT) process [28]

Methodological issue	Obj^a^	Findings
1. Did the study allow a sample size calculation for the definitive trial?	4	Using HbA1c as a primary outcome, the sample size for future randomised controlled trial was estimated to be 492 participants (41 participants from 12 clusters).
2. What factors influenced eligibility and what proportion of those approached were eligible?	1	All diabetes centres approached were eligible. In order for young adult participants to be eligible, they had to meet the inclusion criteria and also have attended a clinic appointment between Oct 2019 and Jan 2020.
3. Was recruitment successful?	1	Recruitment proceeded smoothly in 2 of the 4 centres. Due to differing ethics requirements, there were more barriers to recruitment which led to lower numbers of participants being recruited in the remaining 2 centres.
4. Did eligible participants consent?	1	Consent was obtained successfully in all centres
5. Were participants successfully randomised and did randomisation yield equality in groups?	1	This was a cluster randomised RCT. Randomisation was successful. However, one centre from the control arm left the study due to delays with ethics and the onset of COVID-19. This, along with additional ethics committee requirements around recruitment, meant the numbers of participants in the control arm was small (see CONSORT flow diagram for detail).
6. Were blinding procedures adequate?	1	Not applicable to current study
7. Did participants adhere to the intervention?	1	Young adult participants and support workers filled in the “study delivery checklist” after each appointment. Adherence was generally good. Three consistent deviations were identified: see text for details
8. Was the intervention acceptable to participants?	1	Young adult participants’ perceptions of the agenda-setting tool and support worker were very positive. Their views on Florence were mixed. Staff held similar views on the intervention components but had an additional concern around resourcing of the Support Worker.
9. Was it possible to calculate intervention costs and duration?	3	The implementation cost of the D1 Now intervention over 12 months was estimated at €1281 per young adult for the internal support worker arm and €1804 for the external support worker arm.
10. Were outcome assessments completed?	2	Young adult self-reported outcomes were 100% completed at baseline and 71% at time 2. This second lower rate of completion was likely caused by time 2 data collection happening through the post, rather than in the clinic, due to COVID-19 related restrictions. Young adult clinical outcomes were 100% completed at baseline and time 2, with the exception of HbA1c, which again was impacted by COVID-19 related restrictions.
11. Were outcomes measured the most appropriate outcomes?	2	Yes, outcomes were based on a core outcome set developed for this population [22]. Young adult participants were extremely positive about being asked to complete questionnaires dealing with the psychosocial aspects of living with diabetes.
12. Was retention to the study good?	1	Yes, there was just 12% attrition from baseline to follow-up. Details are in the CONSORT flow diagram.
13. Were the logistics of running a multicentre trial assessed?	1	Yes. Patient recruitment and intervention delivery in a future definitive trial was identified as being resource intensive. The varying requirements of multiple different Research Ethics Committees were also identified as a challenge.
14. Did all components of the protocol work together?	1	Yes, but some modifications are required to move forward to a definitive RCT.

^a^Study objective

## Discussion

Pilot studies are a crucial step in the development of RCTs and can identify problems in procedures and activities that may not have been obvious in the planning stages [30]. Most elements of the D1 Now pilot RCT protocol were implemented smoothly and generated largely positive feedback from the participants involved. However, the ADePT process allowed us to identify a number of feasibility problems relating to (1) the intervention components and (2) outcome measures. Appendices 4 and 5 outline the ADePT processes for each feasibility problem in detail.

### Feasibility issues with intervention components

The first problem identified with the intervention components was use of the agenda-setting tool. As described previously, the agenda-setting tool was designed to be used by the young adult in conjunction with their clinician. However, in practice, the clinician involved did not always complete their portion of the tool. Through qualitative interviews, the problem was identified to be with doctors who had not received the training given at the rollout of the intervention, due to working in a different service at that time. Herner et al. [31] observed a similar finding when trialling the use of diabetes Patient-Reported Outcome Measures (PROMs) in clinical diabetes consultations in Norway. They suggested that when a clinician did not review a PROM during a consultation, it may have been due to a low sense of project ownership, highlighting the need for organisational incentives, facilitation by management and a cultural shift. Based on these findings we propose an alternative approach for the definitive RCT where internal training on the agenda-setting tool is provided quarterly, to align with the changeover of new junior doctors to the diabetes clinics.

A few feasibility problems cropped up with the rollout of the support worker. The biggest of these was the burden of extra work given to the internal support worker and their colleagues. Staff consistently reported that the internal support worker could not sustain the required workload in the long term. However, although the external support worker was a valued addition to the clinical team, there were also concerns about how an external support worker would be resourced outside of a research study context. A similar programme of research which introduced a “Transition Coordinator” to the process of transitioning young adults from paediatrics to young adult services in Canada saw positive results but acknowledged the costly nature of such a role [32]. They suggest that the key elements of the role could be provided by any staff member and do not necessarily need to involve a highly specialised staff member [32]. We included a health economic sub-study as part of our pilot RCT allowing us to quantify costs; this will inform planning for any future definitive RCT of the D1 Now intervention.

Busy clinics with many participants in attendance meant that sometimes young adults were seen by their clinicians before the support worker (contrary to the intervention protocol which requires them to see the support worker first). Further discussion may need to occur to establish how to fully integrate the support worker into the clinic flow. Based on these findings we propose an alternative approach for the definitive RCT where a support worker will only take on a set number of participants per clinic in order for all participants to move through the clinic as required by the study protocol.

The final intervention component of Florence also had some feasibility issues, the most prominent of these being it was not liked by some participants. It also seemed that among those who did like it, very few were using it to its full interactive potential and rather were using it as a passive reminder system. We propose a potential solution for a future RCT of removing Florence from the intervention and collapsing its functions into the support worker role i.e. have the support worker identify goals and barriers to daily self-management with the young adult and set up a reminder system on the young adult’s own device (e.g. smartphone) to target these.

### Feasibility issues with outcomes

There were no feasibility issues with the self-report outcome measures and they were very well received by the participants. The health economics analysis also proved feasible and acceptable, producing a set of cost and QALY results. However, the paper-based mode of data collection did present a problem and led to a lower response rate at the 12-month timepoint. We propose a potential solution for a future RCT of using an electronic questionnaire for data collection which has the added advantage of simplifying data entry.

In terms of the clinical outcome measures, a few feasibility issues were raised. One of these outcomes is “clinic engagement”. We operationalised this as clinic attendance. However, after reflection with clinic staff, it was highlighted that it may have been more meaningful to capture all interactions, such as telephone calls, emails, nurse visits etc. We will operationalise “clinic engagement” this way in the future. The clinical outcome measure which posed the largest problem was HbA1c. The response rate at end of the study was low, mainly due to COVID, but there were some other difficulties which also occurred at baseline which are described in the “Results” section. We propose two possible solutions: the use of dried blood spot collection in the participant’s home (see [33] for a description of this process) and/or funding of dedicated phlebotomy support for a future RCT.

### Limitations

Pragmatism was the driver of some decisions in this pilot RCT due to resource constraints. The first of these was the selection of diabetes centres involved in the study. Rather than approaching all centres in Ireland, centres were selected based on geographical factors which facilitated access by the research team. Another was the added inclusion criterion of attendance at a clinic appointment between October 2019 and January 2021 for young adult participants, which was necessitated to ensure study completion within the allocated timeframe. Ideally, all young adults attending each centre would have been invited to take part. This resulted in some otherwise eligible young adults not being invited, but importantly may also have resulted in missing a cohort of young adults who are not currently engaged with their clinic service. These young adults are a crucial cohort to engage as the HbA1c of those who don’t successfully transition to adult care is estimated to be 1.5% higher than those who remain in medical care [34].

Another limitation is the impact of the COVID-19 pandemic and its associated restrictions on the delivery of the pilot RCT. As outlined in the “Impact of COVID-19” section, this resulted in clinics being moved to a virtual environment for at least 3 months in all centres. The D1 Now intervention is designed for an in-person clinic and it is possible that participants did not receive the full intervention experience during this time. However, our qualitative research captured participants’ views on this and this information will inform a future version of the D1 Now intervention which can be used in both the in-person and virtual environment. Experience with remote consultation during the pandemic suggests that while a return to face-to-face consultations will be welcomed by many, remote consultation has many advantages and is likely to remain in a post-COVID practice environment.

### Conclusions and future directions

Overall the pilot RCT of D1 Now was successful. Despite the emergence of the COVID-19 pandemic mid trial, we successfully recruited and engaged this highly mobile population. This is likely due to the stakeholder-engaged approach that was taken throughout the intervention development and piloting process [3]. The D1 Now Young Adult Panel contributed to all research processes which ensured the young adult voice was heard throughout. The ADePT process facilitated the identification of problems and has allowed the research team to develop modifications to intervention components and research processes that are necessary to ensure the feasibility of a definitive RCT. The most significant modifications as a result of this pilot include removal of one of the intervention components (the interactive messaging system), a move to electronic data collection and engagement of local phlebotomy services. In addition, we hope that the transparent reporting of this process is a valuable addition to the wider pilot and feasibility methodology literature.

## Supplementary Information

**Additional file 1.**

## Data Availability

The data that support the findings of this study are available on request from the corresponding author. The data are not publicly available due to the small numbers involved and privacy restrictions.

## Abbreviations

T1D: Type 1 diabetes
RCT: Randomised controlled trial
ADePT: Decision-making after Pilot and feasibility Trials
PPI: Public and patient involvement
T1C: Type 1 Diabetes Consultation Tool
HbA1c: Haemoglobin A1c
DDS-2: Diabetes Distress Scale-2 item
T1-DDS: Type 1 Diabetes Distress Scale
CONSORT: Consolidated Standards of Reporting Trials
DKA: Diabetic ketoacidosis
QALY: Quality-adjusted life years
PROM: Patient-Reported Outcome Measure
